# Universal Influenza Vaccines, a Dream to Be Realized Soon

**DOI:** 10.3390/v6051974

**Published:** 2014-04-29

**Authors:** Han Zhang, Li Wang, Richard W. Compans, Bao-Zhong Wang

**Affiliations:** Department of Microbiology and Immunology, and Emory Vaccine Center, Emory University School of Medicine, Atlanta, GA 30322, USA; E-Mails: han.zhang@emory.edu (H.Z.); lwang40@emory.edu (L.W.); rcompan@emory.edu (R.W.C.)

**Keywords:** influenza, cross protection, universal influenza vaccine

## Abstract

Due to frequent viral antigenic change, current influenza vaccines need to be re-formulated annually to match the circulating strains for battling seasonal influenza epidemics. These vaccines are also ineffective in preventing occasional outbreaks of new influenza pandemic viruses. All these challenges call for the development of universal influenza vaccines capable of conferring broad cross-protection against multiple subtypes of influenza A viruses. Facilitated by the advancement in modern molecular biology, delicate antigen design becomes one of the most effective factors for fulfilling such goals. Conserved epitopes residing in virus surface proteins including influenza matrix protein 2 and the stalk domain of the hemagglutinin draw general interest for improved antigen design. The present review summarizes the recent progress in such endeavors and also covers the encouraging progress in integrated antigen/adjuvant delivery and controlled release technology that facilitate the development of an affordable universal influenza vaccine.

## 1. Introduction

Approximately 250,000–500,000 deaths are caused by influenza epidemics worldwide yearly, and the death number may be up to millions in a possible influenza pandemic [1,2,3]. The causative pathogen, influenza virus, belongs to the RNA virus family of *Orthomyxoviridae* and can be classified into A, B and C types. Type A virus is the primary pathogen responsible for seasonal epidemics and pandemic outbreaks. The genome of influenza A virus contains eight negative sense single-stranded RNAs encoding multiple viral proteins, including the surface protein hemagglutinin (HA) which is the main antigen required for protective immunity. According to the phylogeny of HA, type A virus can be further divided into 18 HA subtypes [4,5,6]. The 18 HA subtypes fall into two major groups, with the phylogenetic group 1 viruses containing subtypes H1, H2, H5, H6, H8, H9, H11, H12, H13, H16, H17 and H18 while the group 2 includes subtypes H3, H4, H7, H10, H14 and H15 [6,7]. The current trivalent influenza vaccines are formulated with two type A viruses in subtypes H1N1 and H3N2, respectively, and a virus in type B matching the circulating strains. Although current influenza vaccines are effective in battling closely matched viruses, major limitations are the need to produce new vaccines every season, the uncertainty in choice of the correct strains, a slow production process requiring embryonated eggs, as well as the inability to prevent an influenza pandemic or the emergence of a new drift strain. Of these challenges, the hurdles of antigenic drift and shift present the most important focus for influenza vaccine research and development. Driven by the selective pressure of human immunity, the HA gene undergoes frequent genetic mutation leading to the emergence of new virulent strains [8,9]. For these reasons, the seasonal influenza vaccine has to be reformulated annually based on prediction of the upcoming circulating subtypes. Unfortunately, mismatch between formulated vaccines and the prevalent strains indeed happens and causes severe illness and economic burden [10]. Moreover, genetic reassortment between different subtypes of influenza viruses concurrently infecting the same host can result in novel unexpected viruses that may cause pandemics [11]. A non-human influenza virus may also acquire the capacity for transmission in humans. Because of the frequent infection by highly pathogenic avian influenza A (HPAI) H5N1 in humans in recent years, and the recent outbreak of human infection by a novel avian influenza virus (H7N9) in China [12,13], this concern has become more urgent. All these issues call for the development of a broadly cross-protective influenza vaccine, or universal influenza vaccine, which can confer protection against a broad spectrum of influenza viruses [14,15].

Compared to the traditional inactivated or attenuated influenza virus vaccines, new generations of influenza vaccine employ technologic advances aimed at inducing broad cross protection and enhanced immunogenicity. These advances include rational design of antigens, integrated adjuvant strategies, more efficient delivery platforms and controlled release technology. Advances in such endeavors are discussed below.

## 2. Conserved Antigens with Potential as Universal Influenza Vaccines

The development of efficacious universal influenza vaccines involves antigen designs of highly conserved protein epitopes. Usually these targets are less exposed to the host immune system, and thus stand less immune pressure-derived antigenic changes. These antigens are naturally weakly immunogenic but are expected to elicit immune responses with broader reactivity if they are appropriately presented and sensed by the host immune system [7,16,17]. Currently, conserved epitopes residing in the influenza matrix protein 2 (M2) and the HA stalk region draw general interest as targets for improved antigen design [7,17,18,19]. Further, the same epitopes can be presented in a variety of platforms including soluble proteins with adjuvant, subunit or domain epitopes fused to a carrier protein backbone, virus-like particles (VLPs) and nanoparticles [16,20,21,22,23,24,25]. 

### 2.1. M2e: The Ectodomain of M2

Functioning as a homo-tetrameric ion channel and playing an important role in uncoating virus after viral entry, influenza M2 is expressed as an integral transmembrane protein and consists of 97 amino acids including 24 amino acid residues at the N-terminus which form the ectodomain (M2e) [26,27,28,29]. In human influenza A viruses, M2e is completely conserved in its N-terminal 9 amino acids, and has minor changes in the membrane-proximal region (Table 1) [21,30]. Due to its high conservation among influenza A viruses, M2e has been considered as a promising target for inducing cross protection against different influenza viruses [31,32]. However, M2e-specific immune responses elicited by seasonal vaccines or viral infection are low due to its low immunogenicity resulting from its relatively low epitope density (1–3 copies per virus) and smaller size compared to the other two surface antigens HA and neuraminidase (NA), which may shield M2e from the host immune system. To overcome these limitations, several approaches have been employed to improve M2e immunogenicity. These include: (1) Candidates containing multiple M2e copies to increase the epitope density [14,30,33,34,35,36,37,38,39]; (2) Candidates in which the native tetrameric structure is stabilized and/or presented in a membrane-anchored form in VLPs to simulate the native state of M2e [20,21,36,40]; (3) M2e linked to innate signaling initiators/immune stimulators [20,34,35,36]; (4) M2e assembled in particulate forms, such as VLPs or nanoparticles [20,21,22,36,41,42,43,44]; and (5) Multiple M2e copies presenting major sequences from various strains [38,39]. In one of these studies, vaccine employing human consensus M2e sequence (identical to the sequence of Phi/82, Table 1) elicited immune serum that was highly reactive to Phi/82 H3N2 virus. In contrast, the cross-reactivity to M2e of PR/8, CA/09 or Viet/04 viruses was lower, implying the necessity of incorporating various M2e sequences when designing universal vaccines [21]. Another report showed that candidates containing different sequences of M2e conferred broadened protection [39]. Table 1 lists the human M2e consensus sequence and the differences seen among three human strains and an avian strain. In most studies, integrated strategies combining different approaches induced enhanced M2e-specific immunity with improved protection. Table 2 summarizes some of the important endeavors on M2e-based influenza vaccine studies. Of these, Phase I clinical trials have been completed with two candidates. M2e-HBc in purified protein form or in VLPs is one of the earliest developed candidates [43,45]. It was found that in purified form, the protective efficacy of M2e-HBc conjugate was dependent on NK cells through cell-mediated cytotoxicity [45]. When present in the form of VLPs, its protective immunity was enhanced when adjuvanted with CTA1-DD by intranasal immunization [43]. In a randomized, double-blind, placebo-controlled Phase I clinical trial, the safety and immunogenicity of M2e-HBc VLPs/CTA1-DD (designated ACAM-FLU-ATM) was evaluated in humans, and the results demonstrated that it was promising for further clinical studies. Another candidate, STF2.4×M2e (designated as VAX102 in clinical trials), a fusion protein of M2e with the TLR5-ligand domains from *Salmonella typhimurium flagellin flj B* (STF2), also completed a Phase I clinical trial, and was found to be safe and immunogenic [34,35,46].

Most M2e-based vaccine candidates decrease morbidity in animal models; however, animals showed some illness. This also indicates that other conserved epitopes that can induce neutralizing antibody responses should be combined with M2e to develop a fully protective universal influenza vaccine. Another issue is that some animal models, such as pigs, showed no protection after immunization with M2e vaccines, although robust M2e-specific antibody responses were induced [41,47]. However, it is worth noting that the challenge viruses used in pigs were swine influenza strains while the sequences of M2e in vaccine candidates used for immunization were derived from human or avian viruses [41,47]. In contrast, M2e sequences used for immunization in the other models, including mice, ferrets, rabbits, and rhesus monkeys, were derived from human influenza viruses, and challenge viruses were animal-adapted human viruses [14,33,48]. The M2e sequence differences between the vaccines and challenge viruses may be one of the many reasons for the reduced protection observed in pigs. Including multiple M2e copies presenting viral sequences from various hosts into the vaccine candidates may help to broaden the protective spectrum of M2e-based vaccines [38,39]. These results suggested that extensive challenge studies of protective efficacy against different viruses should be performed to evaluate M2e-based influenza universal vaccines. In addition, combination of M2e with other antigens should be evaluated for augmented neutralizing antibody induction. 

**Table 1:** Sequence difference of M2e from various influenza viruses.

Virus	Subtype	M2e sequence
Human virus M2e consensus	N/A	MSLLTEVETPIRNEWGCRCND
A/Philippines/2/82	H3N2	MSLLTEVETPIRNEWGCRCND
A/Puerto Rico/8/34	H1N1	MSLLTEVETPIRNEWGCRCNG
A/California/04/09	H1N1	MSLLTEVETPTRSEWECRCSD
A/Vietnam/1203/04	H5N1	MSLLTEVETPTRNEWECRCSD

**Table 2:** Summary of M2e-based universal influenza vaccine studies.

Year [ref]	Immunogen	Platform/Adjuvant	Animal model	Protection against viral challenge
1999 [42]	M2e-HBc VLPs	VLPs	Mouse	Partial protection with sickness
2002 [41]	M2e-HBc VLPs or DNA/HBc VLPs	VLPs or DNA/VLPs	Pig	No protection
2003 [48]	M2e	BSA	Rabbit	*In vitro* viral replication-inhibition observed
2003 [30]	M2e-MAPs	MAP	Mouse	Weak protection
2004 [14]	M2 peptide conjugate vaccine	KLH or OMPC	Mouse, ferret, and rhesus monkey	Protection in mouse and ferret challenges
2004 [45]	M2e coupled to HBc	Protein with no adjuvant	Mouse	Weak protection, failed to protect mice from weight loss
2004 [33]	Multiple M2e copies	GST	Mouse, Rabbit	Protected against lethal viral challenge
2006 [43]	M2e-HBc	VLPs/CTA1-DD	Mouse	Protected against lethal challenge
2006 [49]	M2eA	Liposomes	Mouse	Protected against lethal challenge
2008 [44]	PapMV-CP-M2e	VLPs	Mouse	Protected against 4× LD50 WSN/33 strain
2008 [34,35]	STF2.4×M2e	Flagellin fusion	Mouse, Phase I Clinical trial	Mice protected Safe and immunogenic in human use
2008[50]	M2	M2 coupled to RNA phage QβVLP, adjuvanted with CpG	Mouse	Protected against 4× LD_50_ PR8 strain
2009 [51]	M2e-CD154	Salmonella Enteritidis strains	Chicken	Protected against low pathogenic avian influenza (H7N2) but not high pathogenic avian influenza (H5N1)
2010 [52]	M2e-core antigen (woodchuck hepatitis virus)	Salmonella Enteritidis strains	Mouse	Against low dose viral challenge with A/WSN/33
2010 [53]	Pam2Cys	Lipopeptide	Mouse	Weak protection
2011[40]	Tetra-M2e	Nanoparticles	Chicken	Protection against low pathogenic avian influenza H5N2
2012 [54]	M2e-viral capsid protein fusion	VLPs	Mouse	Protected against 4× LD_50_ PR8 strain
2012 [20,36]	4.M2e-tFliC	VLPs	Mouse	Heterosubtypic protection
2012 [37]	4× M2e.HSP70c	4× M2e.HSP70c	Mouse	Broad protection against H1, H3, H9 viruses
2013 [38,39]	M2e × 5	VLPs	Mouse	Broad protection
2013 [22]	M2e-AuNP	Nanoparticles/CpG	Mouse	Heterosubtypic protection
2013 [20]	Tetrameric M2e	VLPs	Mouse	Heterosubtypic protection
2013 [21]	Tetrameric M2e	Nanoparticles	Mouse	Heterosubtypic protection
2014 [16]	4.M2e-tFliC	Microneedles	Mouse	Heterosubtypic protection

### 2.2. HA Stalk Domain

HA is the major influenza antigen inducing neutralizing antibody responses during vaccination and viral infection. Neutralizing antibodies are mainly raised against the membrane-distal head domain, which is subjected to frequent antigenic changes and thus is highly variable among different subtypes. In comparison, the sequence of the membrane-proximal stalk domain is highly conserved among viruses belonging to the same phylogenic group, thus presenting a promising target for universal vaccine design [15,55]. A growing body of evidence has emerged to support the idea that a stalk-based vaccine is capable of eliciting immune responses with broad-cross protection efficacy. These studies include: (1) Multiple monoclonal antibodies (mAbs) were discovered in human B cell-derived libraries. The spectrum of reactivity and neutralizing protection conferred by these mAbs in mouse models extended to a broad array of viruses within individual phylogenetic groups (mAbs CR6261 and F10 for group 1, CR8020 for group 2) or across both groups 1 and 2 (mAb FI6) [56,57,58,59]. (2) Neutralizing anti-stalk antibodies are elicited during seasonal influenza infection but at relatively low levels. These antibodies have been shown to be possibly boosted during the 2009 pandemic, and have been hypothesized to contribute to the subsequent extinction of circulating seasonal strains [60]. This finding further implies that anti-stalk antibodies are effective not only in a mouse model but also in the human population, suggesting the feasibility to develop stalk-based universal vaccines for humans [60,61]. (3) Anti-stalk antibodies can provide protection through passive transfer [57,62,63,64]. Moreover, a recent study showed that intranasal gene delivery of adeno-associated virus vectors (expressing the mAb FI6) to the airway epithelial cells in mice and ferrets elicits broad cross protection against multiple pandemic H1N1 and H5N1 strains [65].

Because the HA stalk domain is shielded from the immune system by the immuno-dominant head domain during natural infection or conventional influenza vaccination, augmented exposure of the stalk domain to the host immune system through antigen design and selected vaccination regimens are crucial. Current strategies employed for stalk-oriented antigen design include the following: (1) Truncated HA that lacks the globular head domain. In this approach the designed headless stalk domain, as a conformational epitope, is required to maintain its appropriate conformation (pre-fusion) despite the absence of head domain. One of the most critical issues here is how to stabilize the headless HA, which is predisposed to instability. Some progress has been made on optimization of antigen design and construction, which were facilitated by the use of recombinant protein expression and protein minimization methods [66,67]. These resulting vaccines only conferred protection against lethal homologous virus challenge. It was found that when expressed in the form of VLPs in a mammalian system, the headless stalk domain was only expressed at low levels and the conformational stability of the pre-fusion stalk domain was not confirmed [68]. (2) Short peptide epitopes (including the fusion peptide and A-helix) from the stalk domain. These selected peptides can be fused with carrier proteins (for instance, keyhole limpet hemocyanin) for improved antigen presentation and enhanced immunogenicity based on the adjuvant function of carrier proteins. One recent report showed the competence of this fusion protein approach in conferring broad protection against influenza subtypes from both groups 1 and 2 [64]. (3) Sequential vaccination with a panel of recombinant chimeric HA (cHA) proteins each containing an identical stalk domain in pair with head subunits from different influenza virus subtypes [69,70]. In this case, cHAs retained an intact structure resembling the wild type HA, but selectively boosted immune responses against the stalk domain [60,68]. In fact, sequential exposure to antigenically divergent wild type HAs can also induce broadly reactive antibodies specific to stalk domains [64]. Previous exposure to distant homosubtypic viruses prior to vaccination actually enhances stalk-directed immune response in ferrets [61]. In addition, vaccination regimens including DNA-priming followed by a heterologous protein/inactivated virus/replication-defective adenovirus vector-boost were found to elicit high titers of stalk-specific antibody responses [61,71,72]. (4) Modification of the head domain to mitigate its immune−dominance, for instance by N-linked glycosylation modification of immune-dominant antigenic sites in the head domain. It has been demonstrated that the hyper-glycosylated HA protein induced higher (compared to wild type HA) titers of stalk-directed antibodies which react with a panel of both heterologous and heterosubtypic viruses [73]. On the other hand, it was found that vaccination of monoglycosylated H1N1 HA (HA_mg_) induced significantly enhanced cross-protection against multiple homosubtypic strains compared to both fully glycosylated (HA_fg_) and unglycosylated HAs (HA_ug_). Additional data suggested that HA_mg_ elicited enhanced CD8^+^ cytotoxicity effects as well as HA−specific cross-protective antibody secretion [74].

Antigens induce antibody responses including both neutralizing and non-neutralizing antibodies. Recent studies demonstrated that non-neutralizing antibodies sometimes can even enhance infectivity [75,76]. The virus-antibody complex can be recognized by Fcγ receptors on macrophages or other types of cells, which may promote endocytosis of virus particle and subsequent viral infection events [77,78,79]. Khurana *et al.* reported that whole inactivated H1N2 virus (WIV-H1N2) vaccination resulted in enhanced vaccine-associated pneumonia and disease after mismatched pH1N1 virus challenge in swine [80]. A short fragment downstream of the fusion peptide in the stalk domain of H1N1 HA was found as the predominant epitope for WIV-H1N2 immune sera. It was suggested that this non−neutralizing anti-stalk antibody may promote H1N1 infection by enhancing H1N1 virus membrane fusion activity. These findings imply the necessity of careful evaluation of non-neutralizing antibody responses and vaccine-enhanced disease during the development of HA stalk-based universal vaccines.

As the primary influenza vaccine targets, conserved domains harbored in the surface proteins HA and M2 have been widely investigated as mentioned above. M2e-based vaccines were shown to elicit non-neutralizing antibody responses, and the protection conferred by these vaccines in animal models is possibly mediated by antibody-dependent cell cytotoxicity (ATCC) responses and NK cell/complement-mediated infected cell elimination mechanisms [31,45,81]. In contrast, neutralizing antibody responses are readily elicited by HA-based vaccines. Further, both M2e- and HA stalk−elicited serum antibodies have been shown to confer protection through passive transfer [21,57,62,63,64]. It is hopeful that vaccine formulations combining HA and M2e epitopes would offer a more effective vaccination approach. 

Besides the progress obtained for M2e and HA stalk-based universal influenza vaccines which is the main topic of the present review, multiple other strategies have also shown their promises. One of these many directions for universal influenza vaccine development is the cellular immunity elicited by highly conserved viral epitopes which reside on various influenza proteins including viral nucleoprotein (NP), matrix protein M1, RNA polymerase subunits PB1 and PB2. The pivotal role of T cell immunity in heterosubtypic protection during natural influenza infection in the human population has been recently reported [82], which implies the urgent necessity for incorporating both B- and T-cell epitopes into a single vaccine for mounting both arms of immunity. An attractive candidate (multimeric-001) is one of such vaccines. Multimeric-001 includes multiple linear epitopes from HA, NP and M1, and has passed a Phase I/II clinical trial investigation showing safety and immunogenicity in humans [83].

## 3. Integrated Adjuvant/Delivery Platforms and Controlled Release Technology Can Contribute to the Development of Universal Influenza Vaccines

In addition to antigen design, integrated adjuvant/delivery platforms and controlled release technology have been employed to enhance the immune response and cross-protective efficacy of influenza vaccines. Of these, VLPs, a nanoscale self-assembling system, are one of the most attractive platforms [20,36,39,42,84,85,86]. By mimicking the organization and conformation of native viruses but lacking the replicative genomic information, VLPs can be produced in heterologous expression systems in large scale, and thus can yield safer and cheaper vaccine candidates [87,88]. Because of the self-assembly feature of VLPs, targeted viral antigens form multimeric complexes displaying a high density of epitopes which simulate the natural structures of viral pathogens. By using molecular biotechnology, non-associated epitopes may be assembled or incorporated into VLPs by genetic modification using different expressing systems. For instance, Neirynck *et al.* fused M2e to the hepatitis B virus core (HBc) to create a fusion gene coding for M2e-HBc; M2e-HBc VLPs were efficiently produced using an *Escherichia coli* (*E. coli*) expression system. Intraperitoneal or intranasal administration of purified M2e-HBc particles in mice provided 90%–100% protection against a lethal virus challenge [42]. The high yield and rapid protein production in the *E. coli* system enables a significantly shortened vaccine production time-line and improves preparedness against unexpected pandemics. A novel vaccine, with an *E. coli* expressed HA globular domain conjugated with the bacteriophage Qβ VLPs, has been recently demonstrated to elicit high titers of antigen-specific antibody and Th1 biased T-cell responses, and can confer protection against highly drifted homosubtypic strains [89,90]. By attaching a tetramerization sequence and transmembrane/cytoplasmic domains to M2e and expressing the resulting construct in insect cells, we found that M2e can be presented as tetramers, the natural structure of M2e in the virion, on the surfaces of influenza M1 VLPs. The resulting VLPs conferred cross-protection in mice [20]. Additionally, by employing a mammalian expression system, HA stalk domains were incorporated into HIV Gag-derived VLPs and conferred protection against influenza viruses [66]. Because of the relatively large surface of VLPs, both antigens and immune stimulators (protein adjuvants) may be co−incorporated into chimeric VLPs (cVLPs). For instance, M2e and modified flagellin, or their fusion proteins, have been co-incorporated into cVLPs for enhanced cross protection [20,36]. These universal influenza vaccine candidates conferred broadened protection when compared to their soluble counterparts. An advantage of co-delivery of both antigens and adjuvants into the same immune cells by cVLPs is that antigen-specific immune responses can be enhanced due to the innate signaling co−stimulated by adjuvant molecules in cVLPs. Since the size and conformation of these particles are similar to the intact native virions which the immune system evolved to battle, VLPs have been demonstrated to be highly immunogenic as a new influenza vaccine platform [91,92,93,94,95]. Moreover, VLPs can enter both major histocompatibility complex (MHC) class I and class II antigen processing pathways in antigen presenting cells (APCs), eliciting both humoral and cellular immune responses [95,96,97]. In conclusion, as an integral platform, VLPs provide great potential for the development of universal influenza vaccines. 

Being assembled in nano scale with controlled antigen release, nanoparticles exhibit adjuvant effects and stimulate APCs upon binding and/or internalization [98,99,100], and have been employed to deliver influenza vaccine for enhanced immune protection [40,101,102]. However, in many cases the amount of antigen loaded into nanoparticles was low due to the presence of a polymer core, and the process by which the particle is prepared can damage or unfold the antigen [98]. Newly developed nanoparticles offer the hope to overcome these limitations [103]. Such nanoparticles can be assembled from proteins under mild conditions and are minimally cross-linked with reversible cross-linkers to preserve protein function. For instance, novel nanoclusters assembled directly from influenza M2e with no need of an encapsulating agent were shown to maximize antigenic protein load [21]. The gentle fabrication conditions allow the antigens to maintain their native forms. These nanoclusters were found to induce cross-protection against viral challenges with different influenza A subtypes, the 2009 pandemic CA/2009 (H1N1) as well as the Philippines/82 (H3N2) viruses [21]. Recently, self−assembling influenza nanoparticle vaccines have also been produced by fusing HA to ferritin, a protein that naturally forms nanoparticles composed of 24 identical polypeptides [104,105]. The resulting antibodies neutralized H1N1 viruses from 1934 to 2007 and protected ferrets from an unmatched 2007 H1N1 virus challenge. These results indicate that self-assembling nanoparticles can improve the potency and breadth of immunity to influenza, providing a novel platform for development of universal influenza vaccines.

With the development of novel vaccine-delivery technology, painless, simple-to-administer microneedles have also been used as new platforms for influenza vaccine delivery and enhanced immune protection. Microneedle arrays are designed to penetrate the stratum corneum, the outer layer of the skin, and deposit a vaccine or drug into the epidermis and dermis [106,107,108]. Because the skin contains various kinds of immune cells (including keratinocytes and Langerhans cells (specialized dendritic cells) in the epidermis, dendritic and mast cells in the dermis, and T as well as B cells in the skin-draining lymph nodes), it is an attractive site for the administration of vaccines and immunomodulators [109]. Further, simplified administration of a universal influenza vaccine would greatly reduce the morbidity and mortality from a newly emerged influenza pandemic when general resources such as vaccine production, storage, transportation and healthcare service facilities are limited [16]. Various antigen forms including recombinant antigen-adjuvant fusion proteins, inactivated virus, VLPs or subunit vaccines have been coated on microneedles and shown to induce improved protective immunity by skin vaccination [16,110,111,112]. Skin vaccination by microneedles delivering the fusion protein 4.M2e-tFliC induced heterosubtypic protection in mice [16]. The dose−sparing effect of microneedle delivery seen in this study provides a great benefit for preventing an emerging influenza pandemic because the available vaccine production capacity can yield more vaccine doses. The broadened protection was also observed after co-immunization of A/Puerto Rico/8/1934 (A/PR8, H1N1) HA DNA together with inactivated virus by coating on a microneedle patch [113]. With the advantages for painless administration, safety and storage, enhanced stability in dry formulations, and suitability for rapid global distribution in response to possible outbreaks of pandemic influenza, skin vaccination using microneedle-based delivery of integrated influenza antigen/adjuvant compositions is a promising approach for an easy-to-administer universal influenza vaccine.

## 4. Conclusions

In summary, universal influenza vaccine development attracts considerable attention due to its great significance and potential for public health. The urgent need for full preparedness against seasonal and pandemic influenza necessitates not only delicate antigen design and an efficient vaccine delivery system, but also in-depth understanding of the mechanisms involved in the elicited immunity. The exciting progress discussed above holds great promise for a full spectrum of universal protection through this new generation of vaccines. The final fulfillment of an affordable and efficacious universal influenza vaccine will require continuing progress in multiple aspects involved in both preclinical and clinical studies, and may ultimately eliminate the threat to public health from influenza viruses.
